# Use of Natural Biomolecules in Animal Feed to Enhance Livestock Reproduction

**DOI:** 10.3390/ijms26052328

**Published:** 2025-03-05

**Authors:** Ikram BenSouf, Mariem Saidani, Asma Maazoun, Bochra Bejaoui, Manel Ben Larbi, Naceur M’Hamdi, Hebib Aggad, Nicolas Joly, Janne Rojas, Marielba Morillo, Patrick Martin

**Affiliations:** 1 Animal and Food Resources Laboratory (LRAA), National Agronomic Institute of Tunisia, University of Carthage, 43 Av. Charles Nicolle, Tunis 1082, Tunisia; bensoufikram2@gmail.com (I.B.); naceur_mhamdi@yahoo.fr (N.M.); 2 Research Unit of Biodiversity and Resource Development in Mountain Areas of Tunisia, UR17AGR14, Higher School of Agriculture of Mateur, University of Carthage, Tunis 1082, Tunisia; mariiem.saidani@gmail.com (M.S.); arbi_mana@yahoo.fr (M.B.L.); 3 Horticultural Science Laboratory, LR13AGR01, National Agronomic Institute of Tunisia, University of Carthage, 43 Av. Charles Nicolle, Tunis 1082, Tunisia; asmamaazoun@gmail.com; 4 Laboratory of Useful Materials, National Institute of Research and Pysico-Chemical Analysis (INRAP), Technopark of Sidi Thabet, Ariana 2020, Tunisia; bochrabej@yahoo.fr; 5 Department of Chemistry, Faculty of Sciences of Bizerte, Zarzouna, University of Carthage, Bizerte 7021, Tunisia; 6 Laboratory of Hygiene and Animal Pathology, Institute of Veterinary Science, University of Tiaret, Route d’Alger BP 78, Tiaret 14000, Algeria; h_aggad@yahoo.com; 7 Unité Transformations &Agroressources, ULR7519, Université d’Artois, UniLaSalle, F-62408 Béthune, France; nicolas.joly@univ-artois.fr; 8 Biomoléculas Orgánicas Research Group, Faculty of Pharmacy and Bioanalysis, University of Los Andes, Mérida 5101, Venezuela; janne.rojas24@gmail.com; 9 Ecology and Nutrition Research Group, Faculty of Pharmacy and Bioanalysis, University of Los Andes, Mérida 5101, Venezuela; marimorillo44@gmail.com

**Keywords:** feed additives, natural biomolecules, livestock reproduction, sperm quality

## Abstract

Feed additives are crucial in livestock production, enhancing performance, health, and reproductive efficiency. Recently, there has been a shift toward natural biomolecules as feed additives, specifically targeting improved reproductive outcomes and sperm quality. This transition arises from concerns about antibiotic misuse, antimicrobial resistance, and consumer preferences for eco-friendly products, along with the superior bioavailability, lower toxicity, and reduced environmental impact of natural biomolecules compared to synthetic alternatives. Collaboration among researchers, veterinarians, nutritionists, and regulators is essential to ensure safe and effective livestock management. The review explores advancements in using vital biomolecules in reproductive processes, including plant-derived bioactives such as phytochemicals and antioxidants. It investigates not only the mechanisms but also the intricate interactions of these compounds with animals’ hormonal and physiological systems. Additionally, the review critically assesses challenges and prospects related to incorporating natural biomolecules into livestock practices. The potential benefits include enhanced reproductive efficiency and improved sperm quality. However, successful implementation requires understanding factors like precise dosing, potential interactions, and long-term health impacts. Overall, this comprehensive review highlights recent research, technological strides, and the future potential of integrating natural biomolecules into animal diets.

## 1. Introduction

Livestock production is pivotal to safeguarding global food security and bolstering economic stability. The livestock sector contributes approximately 40% of the global agricultural output, supporting the livelihoods and food security of nearly 1.3 billion people, while in developing countries, it accounts for 15 to 80% of agricultural gross domestic product (GDP) [1]. Within this industry, optimizing reproductive performance is a key determinant of sustainability and profitability [2]. Therefore, effective reproductive management strategies are essential to increase animal populations’ productivity [3,4]. In addition, the nutritional status of animals plays a vital role in regulating reproductive processes, including sperm quality [5,6]. Natural biomolecules in feed ingredients have gained popularity as animal feed additives in recent years due to their potential to enhance animal reproductive efficiency and sperm quality [5,7]. Furthermore, using natural biomolecules as feed additives aligns with the growing demand for sustainable and environmentally friendly livestock production practices [8,9]. As consumers become more conscious of the origin and quality of animal products, adopting natural feed additives can enhance the overall image of the livestock industry and its commitment to animal welfare and eco-friendly practices [10,11]. These biomolecules, obtained from plants such as Ashwagandha (*Withania somnifera*), Maca (*Lepidium meyenii*), and Horny Goat Weed (*Epimedium* spp.), microbes, and marine animals, have demonstrated encouraging results in maintaining reproductive health and fertility in livestock species [12,13]. Moreover, a key advantage of utilizing natural biomolecules as feed additives is their capacity to provide a safer and more environmentally sustainable substitute for synthetic additives [14,15]. These biomolecules are frequently high in bioactive substances such as polyphenols, flavonoids, essential fatty acids, and antioxidants, which can improve reproductive performance and sperm quality [12,13,16,17,18]. Specific polyphenols derived from plants, such as resveratrol from grape (*Vitis vinifera*), epigallocatechin gallate from green tea (*Camellia sinensis*), and ellagic acid from pomegranate (*Punica granatum*), for instance, have been documented to alleviate the ovarian function and enhance the synthesis of sex hormones in female animals. This, in turn, contributes to enhanced estrus synchronization and elevated conception rates [12]. Additionally, these biomolecules have demonstrated potential in reducing oxidative stress and inflammation, which may enhance sperm quality and motility in males [19]. However, while the potential of natural biomolecules as feed additives for livestock is promising, further research is needed to understand their mechanisms of action and long-term effects [16]. By harnessing the bioactive compounds in these biomolecules, the livestock industry can promote sustainable practices and meet the demands of conscious consumers seeking high-quality animal products [11]. This literature review examines the potential and innovation of utilizing natural biomolecules as feed additives and their effects on reproduction and sperm quality in livestock.

## 2. Effects of Natural Biomolecules on Female Reproduction and Sperm Quality

Scientists have investigated several natural macromolecule’s effects on animal reproductive performance and sperm quality [5,15,20,21]. These biomolecules have promising effects in boosting livestock fertility, sperm viability, and overall reproductive efficiency [5]. Several plant-derived chemicals have been investigated for their ability to improve animal reproduction. Plant estrogens, such as genistein and daidzein, have improved male fertility [22,23]. However, Hashem and Soltan [24] reported that overexposure to phytoestrogen-containing diets in livestock has been associated with both negative and positive effects. The consumption of phytoestrogens has been shown to result in embryonic loss, poor semen quality, progesterone deficiency, and inhibition of estrus, dependent on the dosage and reproductive status of the animal [25]. Furthermore, flavonoids such as quercetin and resveratrol have been linked to increased sperm motility and viability in males [26]. For example, casein-derived peptides (casokinins and beta-casomorphins) have been related to better ovarian function and embryo development in female livestock [27]. Similarly, certain amino acids (glutamine, proline, and leucine) and peptides derived from animal tissues (arginine, tyrosine, and glycine) have been found to improve male sperm production and quality [28]. Probiotics and prebiotics generated from microbial sources have gained popularity due to their ability to improve animal reproduction in species like cattle and pigs [29,30]. By modifying the microbiota in the intestines and boosting nutritional absorption, probiotic administration has been shown to improve reproductive performance in both female and male animals in sheep and goats [31,32]. Furthermore, prebiotics, such as fructooligosaccharides, have been linked to improved sperm parameters and reproductive outcomes in boars [33]. These natural biomolecules represent a possible alternative to synthetic chemicals and hormones, which can be harmful to animal health and the environment. More study is needed, however, to optimize dosage, timing, and administration mechanisms for optimal usage in livestock production systems.

### 2.1. Amino Acids

**Figure 1:**
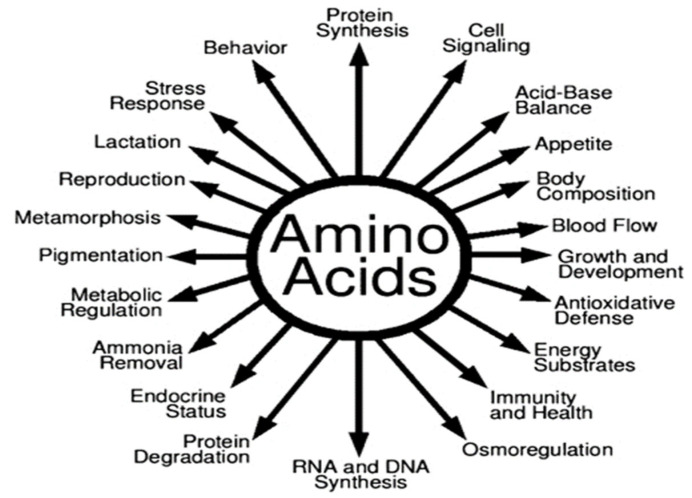
Roles of AAs in nutrition and regulatory functions in human and animal cells [35].

Amino acids (AAs) are essential components of protein synthesis and are involved in a variety of physiological activities, including reproduction [34]. Based on nitrogen balance or growth, amino acids were traditionally classified as nutritionally essential or non-essential for animals and humans [35]. The role of amino acids extends beyond protein synthesis, with several amino acids playing vital roles in reproductive processes. Figure 1 shows several roles that AAs fulfill [34,36]. Amino acid supplementation offers a promising Glycineng approach to optimizing reproductive performance in livestock [37]. These protein building blocks are involved in a variety of physiological processes such as hormone synthesis, gamete formation, and embryo growth [38].

#### 2.1.1. Essential Amino Acids (EAAs)

Leucine and tryptophan, essential amino acids, play prominent roles in stimulating protein synthesis, inhibiting proteolysis (leucine), and modulating various functions, including neurological and immunological functions, through metabolites like serotonin and melatonin, as demonstrated by Wu [35]. Several studies have shown that particular amino acids favorably influence reproductive parameters in diverse animal species. Lysine supplementation has been linked to increased ovulation and conception rates in sheep [39] and better embryo development in cattle [40]. Methionine is required for oocyte quality and early embryonic development in dairy cows and, thus, it has been linked to higher conception rates and lower embryonic losses [41]. Taurine is implicated in ram sperm function and maturation [42]. Taurine supplementation has been shown in numerous animals to increase sperm motility and fertility. According to Bahrami et al. [43], glutamine is necessary to develop cow follicles and embryos. Glutamate supplementation was reported to boost conception rates and embryonic survival. Glycine is required for sows to continue reproducing [44].

#### 2.1.2. Non-Essential Aminoacids (NEAAs)

Non-essential amino acids such as proline, glycine, arginine, alanine, and serine have been found to improve the quality of animal sperm. These amino acids have been associated with benefits in numerous livestock indicators like sperm motility, viability, membrane integrity, and DNA integrity. Several studies have shown that supplementing animal diets with non-essential amino acids can improve sperm quality. Smith et al. [45] reported that supplementing boar diets with proline enhanced sperm motility and viability, resulting in better fertility rates. Shakouri et al. [46] also investigated the effects of glycine supplementation on bull sperm quality. Supplementing glycine increases sperm morphology, membrane integrity, and mitochondrial activity, all of which are critical factors in sperm viability and fertility. Arginine supplementation has been demonstrated to improve sperm quality, motility, and antioxidant capability, which contributed to higher reproductive performance in both boars and bulls [47]. The effects of non-essential amino acids on livestock sperm quality are summarized in Table 1.

#### 2.1.3. Combination of Amino Acids

Certain amino acid combinations appear to benefit both male and female cattle reproduction. Combining particular amino acids may have synergistic effects on fertility in rare situations. According to research, giving female animals the right combination of necessary amino acids in their feed can improve ovulation and conception rates [57,58]. Specifically, studies have demonstrated that optimizing dietary amino acid profiles can support ovarian function and enhance reproductive outcomes [59]. A combination of amino acids considerably impacts acrosome integrity [60]. A study on dairy cows showed that a combination of arginine and methionine supplementation improved oocyte quality and early embryonic development, resulting in higher pregnancy rates [61]. The combination of arginine and lysine has been associated with improved oocyte quality and increased embryo development, leading to enhanced fertility [62]. For males, the association of carnitine and glutamine has been reported to enhance sperm motility and viability, thereby increasing the chances of successful fertilization [63,64]. An adequate mix of arginine, ornithine, and tryptophan intake has been shown to positively influence the release of reproductive hormones such as luteinizing hormone (LH) and follicle-stimulating hormone (FSH), resulting in improved estrus cycles and fertility rates [65,66]. Amino acids, particularly branched-chain amino acids (BCAAs) such as leucine, isoleucine, and valine, have been reported to improve stress-induced fertility by modulating the hypothalamic–pituitary–adrenal (HPA) axis [67]. The right balance of necessary amino acids can increase female ovulation and fertilization rates, male sperm quality, hormonal balance, and reproductive stress.

### 2.2. Fatty Acids

Fatty acids are key dietary components that may considerably impact animal reproductive performance [68,69] as described in Table 2. The consumption of various fatty acids can affect the generation and metabolism of reproductive hormones as well as the properties of sperm. Polyunsaturated fatty acids (PUFAs), have been demonstrated to affect the production and synthesis of reproductive hormones in livestock [70,71]. Omega-3 fatty acids, such as docosahexaenoic acid (DHA) and eicosapentaenoic acid (EPA), have been linked to increased prostaglandin synthesis, which is required for the beginning and maintenance of estrus and ovulation in females [72]. Excessive consumption of certain types of fatty acids, such as trans-fatty acids, has been linked to disrupted reproductive cycles and decreased fertility in both males and females [73]. They are also important in determining the quality and properties of sperm in males. According to Abdelatty et al. [74], the composition of fatty acids in the diet can change the lipid content of sperm membranes, which in turn influences sperm motility, viability, and overall fertility. Incorporating omega-3 fatty acids into the diet has been linked to improved sperm quality, including increased motility and shape, in a variety of livestock species [75]. An imbalance in the ratio of omega-3 to omega-6 fatty acids in the diet, on the other hand, may result in increased generation of reactive oxygen species (ROS) in sperm, leading to oxidative stress and possible DNA damage [76]. Overall, the proper balance and type of fatty acids in cattle diets are critical for maintaining normal reproductive hormone levels and sperm characteristics, which ultimately affect fertility and reproductive success.

### 2.3. The Potential of Conjugated Linoleic Acid (CLA) on Embryo Development and Fertility

Conjugated linoleic acid (CLA) is a polyunsaturated fatty acid with potential health advantages, and effects on embryo development and fertility [85,86]. Furthermore, CLA’s antioxidant properties have been proposed to protect embryos from oxidative stress [87]. In females, CLA supplementation has been associated with enhanced ovulation and hormonal regulation, potentially benefiting females with fertility issues [88]. In males, CLA has been reported to improve sperm quality by enhancing motility and viability [89]. These findings imply that CLA may be useful in promoting fertility in both sexes. However, while multiple studies suggest that CLA has a favorable effect on embryo development and fertility, the mechanisms and long-term benefits of CLA supplementation in animals are not entirely known. More research is needed to determine the ideal CLA dosages and any negative effects for these specific uses.

### 2.4. Antioxidants

Antioxidants have been extensively studied for their potential impact on oocyte quality and embryo development in assisted reproductive technologies (ARTs) [90]. Oocytes and embryos are highly sensitive to oxidative stress, which can lead to cellular damage and impaired development [91]. Antioxidants play a crucial role in neutralizing reactive oxygen species (ROS) and protecting oocytes and embryos from oxidative damage, thus improving their overall quality and developmental potential [7]. Several studies have investigated the effects of various antioxidants on oocyte quality and embryo development [7,90,91]. Resveratrol supplementation significantly improved bovine oocyte maturation rates and enhanced embryonic development [92].

#### 2.4.1. Selenium

Selenium is an essential trace element that is required for the proper action of antioxidant enzymes such as glutathione peroxidase. Selenium supplementation has been shown to improve livestock sperm quality. In a study on rams, selenium supplementation increased sperm motility and viability while decreasing ROS levels [93]. Similarly, selenium treatment improved the sperm shape and mitochondrial function in bulls, resulting in increased fertility [94].

#### 2.4.2. Other Antioxidants

Other antioxidants like zinc, copper, and coenzyme Q10 have been studied for their possible function in increasing sperm quality in livestock (Table 3). Zinc, for example, is important in enzymatic antioxidant defense mechanisms and has been linked to increased sperm motility and chromatin integrity in wild boars [95]. Copper supplementation, on the other hand, has been found to improve antioxidant capacity and sperm quality in rams [96]. Coenzyme Q10, an electron transporter in the mitochondrial respiratory chain, has been proven in animal tests to reduce oxidative stress and improve sperm parameters [97]. Selenium improves oocyte quality and reproductive performance by acting as an antioxidant, reducing oxidative stress in oocytes and embryos, and promoting hormonal balance for ovulation and pregnancy in cattle [98]. It enhances the sperm quality in males and reduces embryonic mortality by preventing oxidative damage during early development. Studies have shown that selenium supplementation improves conception rates and offspring survival in livestock, such as dairy cows, sheep, and goats [99,100].

#### 2.4.3. Interaction Between Different Antioxidants and Their Cumulative Effects on Reproduction

A combination of various antioxidants has been found to have a significant impact on animal reproduction. When coupled synergistically with antioxidants such as vitamin E and selenium, zinc and copper, coenzyme Q10 and L-carnitine, melatonin and vitamin C, and resveratrol and quercetin, they have demonstrated hopeful effects in boosting reproductive performance in several livestock species. Assunção et al. [107] found that supplementing dairy cows with vitamin E and selenium synergistically boosted reproductive performance. The antioxidants collaborated to lower oxidative damage and boost fertility rates. Combining these antioxidants demonstrated potential as an effective technique for improving avian reproductive function [108,109]. Melatonin and vitamin C co-administration were found to have a favorable impact on sheep reproductive performance in a study by D’occhio et al. [110]. The ovarian function improved, conception rates increased, and embryonic losses decreased. Murphy et al. [111] demonstrated that combining resveratrol and quercetin supplementation improved beef cow reproductive efficiency. These antioxidants show promise in terms of reducing oxidative stress and boosting reproductive health.

### 2.5. Vitamins

Vitamin supplementation plays a crucial role in improving reproductive outcomes in various livestock species [112,113]. Adequate vitamin intake has been shown to positively influence fertility, conception rates, and overall reproductive performance.

#### 2.5.1. Vitamin A

Vitamin A, an essential fat-soluble vitamin, significantly improves reproductive processes. According to research, enough vitamin A supplementation improves follicular growth, oocyte quality, and embryo survival in cattle and sheep [113,114].

#### 2.5.2. Vitamin E

Vitamin E, a powerful antioxidant, has been well-researched for its potential to preserve sperm cells from oxidative damage. In cattle, vitamin E supplementation increases sperm quality and motility while also protecting sperm cells from oxidative stress [112,115]. Furthermore, vitamin E has been demonstrated to improve ram sperm fertility by lowering lipid peroxidation and DNA breakage [116]. Vitamin E has been linked to greater lambing rates and lower pregnancy losses in sheep [117].

#### 2.5.3. Vitamin C

Vitamin C, a water-soluble antioxidant, has been shown to scavenge ROS and protect sperm cells from their detrimental effects. Vitamin C supplementation has been linked to better sperm quality indices in boars, including higher sperm concentration and motility. Furthermore, vitamin C has been demonstrated to protect sperm DNA from oxidative stress, which is critical for proper fertilization and embryonic development [118].

#### 2.5.4. Vitamin D

Vitamin D has emerged as an important regulator of animal reproductive systems. Adequate vitamin D levels in cattle and sheep have been associated with higher fertility and conception rates [119].

#### 2.5.5. Vitamins B

Several B vitamins play important roles in animal reproduction. Vitamin B12 stands out among water-soluble vitamins for its involvement in cattle reproduction. Vitamin B12 supplementation has been shown to improve sperm quality, ovarian function, and embryo development in cattle [120]. Similarly, vitamin B12 has been linked to enhanced oocyte quality and conception rates in sheep [121]. Other B vitamins also play essential roles: Vitamin B9 (folate) is vital for DNA synthesis and cell division, influencing embryonic development and fertility in cattle and sheep [122]. Vitamin B6 (pyridoxine) supports hormone regulation and protein metabolism, with deficiencies linked to reduced fertility and poor embryo development in cows [123]. In poultry, B6 deficiency in breeder diets affects methionine metabolism in embryos, reducing hepatic S-adenosylmethionine concentrations and altering polyamine levels [124]. Vitamin B2 (riboflavin) supports ovarian health and egg quality, and its deficiency can impair conception rates and embryo survival in poultry [125,126]. Vitamin B1 (thiamine) deficiency in turkey breeders can lead to polyneuritis symptoms in newly hatched poults, which can be resolved by supplementing the breeder’s diet with 2 mg/kg of thiamine [127]. Maternal thiamine supplementation in broiler chickens increases heart thiamine content and α-ketoglutarate dehydrogenase activity in day-old chicks [128].

### 2.6. Plant Extracts

Plant extracts have been studied for their ability to boost animal reproductive efficiency. Several phytochemicals found in these extracts have been shown to significantly alter reproductive parameters such as fertility, libido, and general reproductive health [3]. Plant extracts’ effects on ruminant fertility have been observed to vary depending on factors such as dosage, administration duration, and individual animal characteristics [7].

Evans et al. [129] found that fenugreek seed extract had a positive effect on reproductive parameters in sheep and goats, including increased ovulation rates and improved estrus behavior. It may also improve semen quality in rams and bucks. According to Al-Bayati and Al-Mola [130], fenugreek seed extract improves reproductive performance in cows by increasing estrus behavior, follicular development, and conception rates. It may also have a positive impact on the semen quality in male ruminants. Chaste tree extract has been investigated for its ability to regulate estrus and improve fertility in goats. It may help to synchronize estrus cycles and increase conception rates in breeding [131]. Srinivasan et al. [132] found that sainfoin extract improves reproductive performance in sheep and cattle. It may improve ram and bull semen quality, reduce embryonic mortality, and increase conception rates. Malekinejad et al. [133] investigated the potential effects of Dong Quai (*Angelica sinensis*) extract on reproductive hormones and estrus synchronization in cows. It may alter hormone levels and increase reproductive efficiency. Newton et al. [134] investigated the effects of black cohosh (*Actaea racemosa*) extract on goat reproductive hormones and estrus behavior. Saw palmetto (*Serenoa repens*) extract has been studied for its effect on male ruminant fertility. It may improve semen quality and reproductive hormone levels, potentially increasing sperm production and viability [135]. Red clover (*Trifolium pratense*) extract contains phytoestrogens, which may influence reproductive processes in ruminants. It may influence estrus synchronization, ovulation, and fertility in sheep and goats [136].

## 3. Mechanisms of Action of Biomolecules on Sperm Quality and Female Fertility

The mechanisms by which various biomolecules affect ruminant fertility can be complex, influencing many aspects of reproductive physiology such as hormonal balance, ovarian function, uterine health, and sperm quality (Table 4). Understanding the mechanisms of action of biomolecules on ruminant fertility involves delving into the intricate interactions between various plant extracts and the reproductive physiology of sheep, goats, and cows. Flavonoids have been shown to have estrogenic activity. They may act similarly to estrogen hormones, promoting estrous behavior and regulating the estrous cycle in ruminants such as sheep and goats [137]. They interact with estrogen receptors, potentially influencing follicular development and ovulation [138]. Polyphenols have antioxidants that can mitigate oxidative stress and inflammation in ruminant reproductive tissues. They can scavenge free radicals and reduce oxidative stress, which is known to impair reproductive performance in ruminants such as cows. By protecting gametes and embryos from oxidative damage, polyphenols may improve fertility outcomes [139]. Saponins have been found to regulate cholesterol metabolism and steroid hormone production. They may increase the synthesis of reproductive hormones like progesterone, which is essential for cow pregnancy [140]. Essential oils such as oregano (*Origanum vulgare*), thyme (*Thymus vulgaris*), and clove (*Syzygium aromaticum*) have antimicrobial properties. They can inhibit the growth of pathogenic bacteria in the reproductive tract, lowering the risk of uterine infections and increasing fertility in goats [141]. Phytoestrogens share structural similarities with endogenous estrogen hormones. They can bind to estrogen receptors in the reproductive tissues of ruminants such as sheep, influencing follicular development and ovulation [142]. Phytoestrogens can act as agonists or antagonists of estrogen receptors, depending on their concentration and subtype. These compounds may impact reproductive processes, including estrous cycling and fertility [37]. Antioxidants protect cells from oxidative stress, which can harm gametes and reproductive tissues. Plant extracts high in antioxidants, such as vitamin E and selenium, have been shown to improve ruminant fertility by reducing oxidative stress and increasing reproductive performance [143]. Fatty acids play an important role in the production and metabolism of reproductive hormones like progesterone and estrogen. They affect the pulsatile release of gonadotropin-releasing hormone (GnRH) from the hypothalamus, which in turn affects the secretion of luteinizing hormone (LH) and follicle-stimulating hormone (FSH) from the pituitary gland [144].

## 4. Challenges and Opportunities

The use of natural biomolecules to enhance fertility in livestock presents both challenges and opportunities [149]. Natural biomolecules, such as peptides, and proteins, play essential roles in various reproductive processes [67,150]. Harnessing their potential to improve fertility in livestock can lead to more sustainable and efficient production systems.

### 4.1. Challenges

Natural biomolecules may have varying effects on different species or even within the same species due to genetic diversity and physiological differences [151]. Determining the appropriate dosage and administration method for natural biomolecules can be challenging, as factors like age, weight, and hormonal status of the animals need to be considered. Many natural biomolecules have low bioavailability when administered orally, requiring innovative delivery methods to ensure effective uptake and action within the animal’s system [152,153]. Natural biomolecules may interact with other compounds or hormones in the animal’s body, potentially leading to unintended side effects that could harm both the animal and its offspring [154,155].

### 4.2. Opportunities

Natural biomolecules can enhance reproductive efficiency by influencing ovulation, sperm production, and embryo development [156,157]. By optimizing reproductive processes, livestock producers can reduce the number of unproductive animals, leading to more sustainable production systems with decreased resource consumption [69]. Natural biomolecules can aid in preserving the genetic diversity of livestock breeds by improving reproductive success rates and allowing for more controlled breeding programs [158]. Hashem et al. [159] investigated the effect of herbal supplements (Ashwagandha *(Withania somnifera*), Maca *(Lepidium meyenii*), and Horny Goat Weed (*Epimedium genus*) on the genetic diversity of Beetal goats and found that certain herbal additives, like common nettle (*Urtica dioica* L.), common agrimony (*Agrimonia eupatoria)*, plantain (*Plantago lanceolata* L.), and thyme (*Thymus vulgaris*), positively influenced genetic diversity, as evidenced by microsatellite marker analysis.

Toledano-Díaz et al. [160] investigated the effect of probiotics on genetic diversity in Holstein cattle. The results indicated that probiotic supplementation improved dairy production efficiency. Bernier et al. [161] studied the impact of herbal feed additives (*Astragali radix*, *Isatis tinctoria Linnaeus*, and *Citri reticulatae pericarpium*) on the genetic diversity of Taiwanese country chickens. Although not directly related to ruminants, this study demonstrates the potential of herbal supplements to improve genetic diversity in livestock populations.

Certain biomolecules such as omega-3 fatty acids, vitamins E and C, methionine, and choline may have positive effects on overall animal health, indirectly contributing to fertility by reducing disease burden and stress [16].

## 5. Technological Advances and Innovations

### 5.1. Nanotechnology

Nanotechnology is a rapidly evolving technology with significant implications for a wide range of therapeutic applications [25,162]. It is capable of addressing a spectrum of animal health and production concerns [25,163]. Nanoparticle-based delivery systems offer tremendous potential for improving the administration of natural biomolecules in animal reproduction [163]. Subsequent research endeavors should prioritize the refinement of nanoparticle design and the comprehensive comprehension of their potential enduring impacts, thereby propelling advancements in the field. The successful administration of natural biomolecules for reproductive interventions faces challenges such as low stability, poor bioavailability, and rapid degradation [21,157]. Nanoparticle-based delivery systems have emerged as promising tools to address these issues and enhance the targeted delivery and controlled release of biomolecules [164]. Diverse natural biomolecules, encompassing hormones, growth factors, and peptides, have been encapsulated within nanoparticles to bolster their stability and selectively target particular tissues or cells integral to the process of reproduction [107]. In their studies, Neeha-et al. [165] explored the use of nanoparticle-based delivery systems for preservation and assisted reproductive techniques (ARTs) in endangered and threatened animal species. They found that natural biomolecules, such as reproductive hormones and cryoprotectants, have been successfully delivered via nanoparticles to support ARTs and improve breeding success in these species. Moreover, in the study of Bernier and Alderman [166] on the application of nanoparticle-based delivery systems in aquatic animal reproduction, nanoparticles were employed to deliver natural biomolecules, such as pheromones and reproductive hormones, to aquatic species, enhancing their reproductive success and supporting conservation efforts.

### 5.2. Microencapsulation

Microencapsulation, a technique that involves encapsulating active compounds within a protective shell, offers several advantages in this context. Firstly, it ensures the controlled release of bioactive compounds, extending their availability in the animal system and potentially leading to prolonged effects [21,157]. This controlled release can help maintain optimal hormone levels, regulate the estrous cycle, and enhance overall reproductive performance [166]. Furthermore, microencapsulation protects sensitive bioactive compounds from degradation in the digestive tract, improving their bioavailability upon ingestion. This is particularly important for compounds with low stability and poor water solubility. By safeguarding these compounds, microencapsulation increases their chances of exerting their desired effects on reproductive physiology. Studies have indicated the positive impacts of microencapsulated plant extracts on livestock reproduction. For instance, microencapsulated phytoestrogens derived from plants like red clover (*Trifolium pratense*) and soybeans (*Glycine max*) have shown potential for regulating estrous cycles and improving fertility in cattle [167,168]. Similarly, microencapsulated essential oils, such as those from thyme (*Thymus vulgaris*) and oregano (*Origanum vulgare*), have demonstrated antimicrobial and antioxidant properties that can support reproductive health in various livestock species [169]. Microencapsulation of bioactive compounds from plant extracts offers a promising avenue for enhancing livestock reproduction.

### 5.3. Nutrigenomics

**Figure 2:**
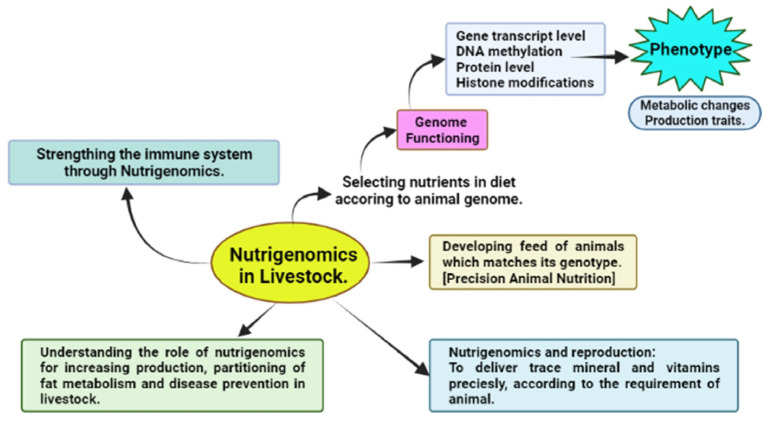
Integration of omic technology in animal nutrition research [172].

Nutrigenomics is a new field that integrates nutrition with molecular technologies to analyze the levels of responses to various nutrients in the body [170]. Attaman et al. [170] and Zhang et al. [171] reported that nutrigenomics focuses on the analysis and comprehension of molecular interactions between dietary bioactive constituents and cellular functioning (Figure 2).

Several studies have revealed that specific nutrients, such as vitamins, minerals, fatty acids, and phytochemicals, act as signaling molecules that interact with transcription factors and epigenetic regulators. For instance, omega-3 fatty acids have been shown to influence gene expression related to fertility, oocyte quality, and embryo development. Nutrigenomics research has shown promising insights into female reproductive health and performance. Studies in mammals have demonstrated that certain dietary components, such as antioxidants and polyphenols, can influence oocyte quality and maturation [173]. Additionally, nutrigenomic interventions during gestation may affect the developmental programming of the offspring’s reproductive system, highlighting the importance of maternal nutrition for future generations [30]. The significance of nutrigenomics in male reproductive performance is of equal importance. Various nutrients have been implicated in sperm quality and function. For example, zinc and selenium have been linked to improved sperm parameters and DNA integrity [174]. Moreover, studies have explored the effects of dietary polyunsaturated fatty acids on spermatogenesis and sperm membrane composition, potentially influencing male fertility [175]. Epigenetic modifications, such as DNA methylation, histone modifications, and microRNAs, play a crucial role in translating nutritional cues into long-lasting changes in gene expression. Nutrigenomics research has highlighted how specific nutrients can alter the epigenetic landscape of reproductive cells, leading to transgenerational effects on fertility and reproductive health [176]. 

### 5.4. Microbiome Modulation

**Figure 3:**
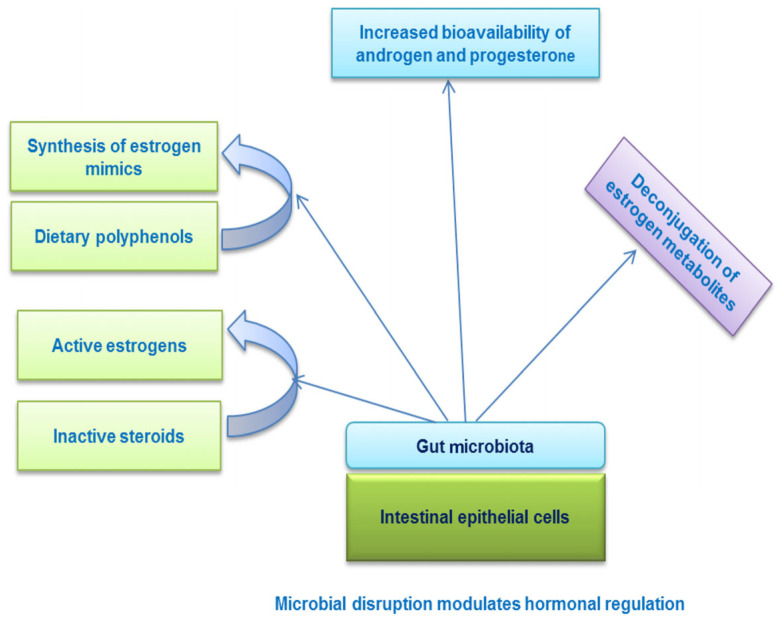
Changes in gut microbiota mediate hormonal regulation [177].

The gut microbiome is an essential regulator of reproductive performance in livestock. The microbiota plays an important role in the reproductive endocrine system by interacting with sex hormones. Hence, any dysbiosis in gut microbiota function influences hormonal activity (Figure 3; [177]).

This suggests that natural biomolecules such as probiotics, prebiotics, plant secondary metabolites, and SCFAs may have promising effects in shaping the gut microbiome to enhance reproduction. Understanding the complex interactions between these biomolecules and the gut microbiome will undoubtedly provide valuable insights into novel strategies for improving livestock reproductive efficiency. Numerous studies have exhibited the favorable impacts of probiotics on gastrointestinal health in livestock, resulting in enhanced nutrient absorption, diminished gut inflammation, and heightened reproductive efficiency [178]. Probiotics have been shown to modulate the gut microbiome by promoting the growth of beneficial bacteria, such as lactobacillus and bifidobacterium while suppressing harmful pathogens. As a result, probiotic supplementation has been associated with enhanced fertility and reproductive success in various livestock species [179,180,181]. By promoting the growth of beneficial bacteria, prebiotics indirectly contribute to improved reproduction in livestock. For instance, fructooligosaccharides (FOSs) have been found to enhance the reproductive performance of sows by influencing the gut microbiome [182]. Studies in ruminant species have shown that certain plant secondary metabolites can improve fertility parameters by reducing pathogenic bacteria and promoting beneficial microorganisms in the gut [183]. Research has shown that short-chain fatty acids (SCFAs) influence ovarian function, embryo development, and fertility in livestock species, making them vital biomolecules for optimizing reproduction [154].

## 6. Conclusions

Optimizing reproductive performance is paramount for sustaining and enhancing livestock production. The reviewed studies encompassed a wide range of topics, including genetics, nutrition, assisted reproductive technologies, and hormonal control. By implementing evidence-based reproductive management strategies, livestock producers can achieve greater efficiency and profitability in their operations, contributing to the overall growth and stability of the livestock industry. The utilization of natural biomolecules as feed additives holds immense promise for enhancing reproduction and sperm quality in livestock. Recent studies have provided valuable insights into the potential benefits of amino acids, fatty acids, antioxidants, vitamins, and plant extracts. Moreover, technological advances such as nanotechnology and microencapsulation offer exciting opportunities to improve the delivery and effectiveness of these biomolecules. Nonetheless, several challenges need to be addressed to fully unlock their potential, encompassing regulatory considerations and economic viability.

## Figures and Tables

**Table 1:** The effects of amino acids on livestock fertility.

Amino Acid	Effect on Male Fertility	Effect on Female Fertility	Reference
Arginine	Improved sperm quality	Enhanced ovulation in sheep	[48,49]
Methionine	Increased sperm count	Improved embryo quality	[50,51]
Lysine	Enhanced sperm motility	Increased conception rate in cows and heifers	[52,53]
Histidine	Improved semen parameters	Regulated estrus cycle in dairy cows	[54,55]
Tryptophan	Enhanced sperm viability	Improved reproductive health in sheep and goats	[56]

**Table 2:** The effects of fatty acids on ruminants’ reproduction.

Fatty Acid	Effects on Male Fertility	Effects on Female Fertility	Reference
Omega-3 Fatty Acids	Improved sperm quality, increased sperm count, and motility.	Enhanced ovarian function, improved oocyte quality, and increased embryo development.	[77]
Omega-6 Fatty Acids	Decreased sperm quality and motility, impaired reproductive performance.	Disrupted ovarian function, decreased oocyte quality, and impaired embryo development.	[78,79]
Saturated Fatty Acids	Negative impact on sperm quality, motility reduced reproductive performance.	Adverse effects on ovarian function, oocyte quality, and embryo development.	[78,80]
Trans Fatty Acids	Detrimental effects on sperm quality and motility.	Disrupted ovarian function, impaired oocyte maturation, and reduced embryo quality.	[81,82]
Monounsaturated Fatty Acids	Mixed effects on sperm quality, moderate influence on fertility.	Variable impact on ovarian function, oocyte quality, and embryo development.	[83,84]

**Table 3:** Effects of antioxidants on farm animals’ Fertility and Semen Quality.

Antioxidant	Effect on Male Fertility	Effect on Female Fertility	Reference
Selenium	Improved sperm quality, increased sperm motility, reduced sperm abnormalities.	Improved oocyte quality, enhanced reproductive performance, and reduced embryonic mortality.	[98,99,100]
Lycopene	Enhanced sperm quality and improved sperm parameters.	Potential improvement in ovarian function, and reduction in oxidative stress.	[101,102,103]
Quercetin	Increased sperm count and improved sperm viability.	Enhanced ovarian function, potential reduction in oxidative stress.	[104,105,106]
Omega-3 Fatty Acids	Improved sperm quality and increased libido.	Improved ovarian function, increased conception rates	[86]

**Table 4:** Mechanisms of action of biomolecules.

Biomolecule	Mechanism of Action	Ruminant Species	Reference
Fatty Acids	Enhance ovarian function, regulate reproductive hormones,	Sheep, Goats,	[145]
	improve oocyte quality, reduce inflammation,	Cows	
	and promote embryo development.		
Amino Acids	Serve as building blocks for protein synthesis,	Sheep, Goats,	[146]
	influencing hormone production and fertility.	Cows	
Antioxidants	Combat oxidative stress, protect gametes and embryos,	Sheep, Goats,	[147]
	improve sperm quality and reproductive efficiency.	Cows	
Plant Extracts	Modulate hormone secretion, enhance uterine environment,	Sheep, Goats,	[148]
	regulate ovarian function, and improve sperm motility.	Cows	[16]
